# Research Progress in Hemicellulose-Based Nanocomposite Film as Food Packaging

**DOI:** 10.3390/polym15040979

**Published:** 2023-02-16

**Authors:** Guoshuai Liu, Kang Shi, Hui Sun

**Affiliations:** 1 College of Chemistry and Materials Engineering, Beijing Technology and Business University, Beijing 100048, China; 2 Beijing Key Laboratory of Quality Evaluation Technology for Hygiene and Safety of Plastics, Beijing Technology and Business University, Beijing 100048, China

**Keywords:** hemicellulose, film, nanomaterial, blending modification

## Abstract

As the main component of agricultural and forestry biomass, hemicellulose has the advantages of having an abundant source, biodegradability, nontoxicity and good biocompatibility. Its application in food packaging has thus become the focus of efficient utilization of biomass resources. However, due to its special molecular structure and physical and chemical characteristics, the mechanical properties and barrier properties of hemicellulose films are not sufficient, and modification for performance enhancement is still a challenge. In the field of food packaging materials preparation, modification of hemicellulose through blending with nanofibers or nanoparticles, both inorganic and organic, has attracted research attention because this approach offers the advantages of efficient improvement in the expected properties and better cost efficiency. In this paper, the composition of hemicellulose, the classification of nanofillers and the research status of hemicellulose-based nanocomposite films are reviewed. The research progress in modification of hemicellulose by using layered silicate, inorganic nanoparticles and organic nanoparticles in food packaging is described. Challenges and outlook of research in hemicellulose-based nanocomposite film in food packaging is discussed.

## 1. Introduction

Food packaging is an essential part of the food industry. Requirements for packaging materials, among others, include protection of food from biological, chemical and physical factors in the circulation of food. Plastics are common food packaging materials because of their low cost, light weight and excellent printability. However, the plastics industry is highly dependent on fossil resources. Excessive use of plastics will inevitably lead to high consumption of nonrenewable fossil resources and environmental pollution [1]. In this context, use of biopolymers as another option is considered helpful in tackling the problems owing to their abundance and renewability [2]. In this regard, lignocellulosic material have been extensively studied [3].

**Figure 1:**
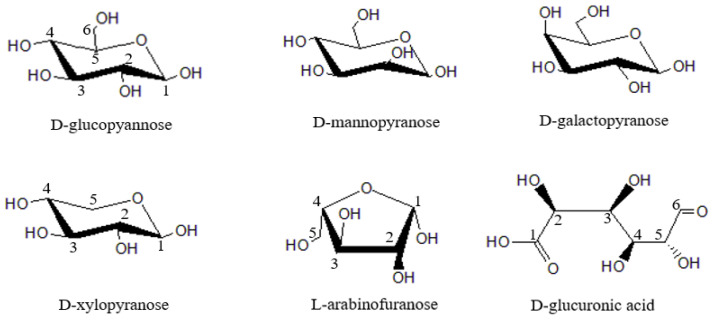
Main structural units of hemicellulose.

Hemicellulos, together with cellulose and lignin, constitute the main components of lignocellulosic biomass. Hemicellulose is an amorphous branched heteropolysaccharide composed of several different neutral and acidic monosaccharides. It has great differences in composition and structure. Basic sugar units of hemicellulose mainly include *D*-xylopyranose, *D*-glucopyannose, *L*-arabinofuranose, *D*-mannopyranose, *D*-galactopyranose and *D*-glucuronic acid (Figure 1) [4]. Hemicellulose has low degree of polymerization, complex structure and strong hydrophilicity. Their conversion into sugar, chemicals, fuel and heat energy has been reported [5].

As a polar polymer, hemicellulose can endow film with good barrier effect against nonpolar molecules (O_2_). Barrier performance and degradability make this polymer promising as potential food packaging. Drawbacks include mechanical strength, hydrophilicity due to high branching degree, low molecular weight and presence of a large number of hydroxyl groups [6]. In development of inexpensive, biodegradable films with properties similar to those of ordinary plastics [7], Modification of hemicellulose for overall performance enhancement (mechanical, barrier, thermal, hydrophobic) is still a challenge. Currently, nanomaterials have been used as additives in a variety of food contact packaging and containers [8] to improve barrier, mechanical and thermal properties. The physical modification of hemicellulose with nanomaterials [9] made the functionalization and utilization of hemicellulose films easier. Xu et al. [10] used green and nontoxic cellulose nanofibers (CNFs) to reinforce hemicellulose/chitosan edible films. These hemicellulose/chitosan edible films with high mechanical strength and flexibility were recommended for food packaging. Zhang et al. [11] prepared functional hemicellulose (HC) film with good mechanical properties, thermo-plasticity and ultraviolet shielding performance by adding nano-ZnO to polyvinyl alcohol (PVA) and hemicellulose. The method improved the application possibility of hemicellulose in the field of food packaging. Therefore, the development of hemicellulose-based nanocomposite films is of great significance for improving the application of hemicellulose-based food packaging and realizing the utilization of biomass resources.

In this paper, the basic compositions and structure of hemicellulose, nanocomposites and nanoparticles used for hemicellulose modification are reviewed. The effects of inorganic and organic nanofillers on the properties of hemicellulose-based composite films and the latest research progress of hemicellulose-based nanocomposite films in the food packaging is highlighted. Finally, the development direction of hemicellulose-based nanocomposite films in modern packaging technology is prospected.

## 2. Hemicellulose

### 2.1. Composition and Structure of Hemicellulose

Hemicellulose, the second-most abundant polysaccharide in plants, is a major component of plant cell walls, accounting for 20~35% of the total weight in cell walls [12]. Hemicellulose is a kind of polyphase and low molecular weight polysaccharide composed of C-6 and C-5 sugar units. Hemicelluloses vary in kinds with complex structure, short molecular chain (degree of polymerization of 80~200) [13], with mostly branched chain structure, and its chemical structure varies greatly in different plants. Hemicelluloses are mainly classified into xylans, mannans, *β*-glucans and xyloglucans based on their structural differences [14]. Hemicelluloses with different structures have very different solubility. Most hemicelluloses can be dissolved in alkaline solution [15,16], and some hemicelluloses with small molecular weight and many branched chains can be dissolved in hot water (Table 1).

**Figure 2:**
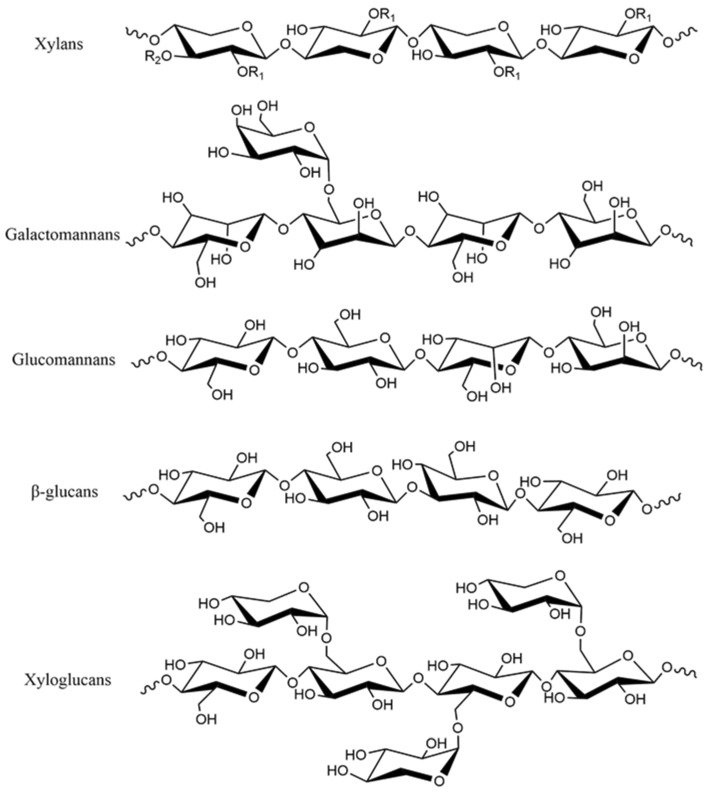
Primary structures of representative hemicellulose. With R1 and R2 are H or different side groups.

The primary structures of different types of hemicellulose are shown in Figure 2 [15,25,26]. Xylan [27,28] is a chain molecule linked by xylose with 1,4-*β* glycosidic bond. Most xylans have acetyl groups, most of which are located at C-3 of xylose and a few at C-4 of xylose. At the same time, 4-*O*-methyl-*α*-D-glucuronic acid is linked to xylose residues. According to the primary structure of xylan in plant tissues, xylan can be divided into homoxylan and heteroxylan. The backbone of homoxylan consists of D-xylopyranose residues linked by *β*-(1→4) or *β*-(1→3) glycosidic linkages, which have structural functions in the cell wall. Heteroxylan includes glucuronoxylans, arabinoxylans and complex heteroxylan. Most glucuronoxylans have single 4-*O*-methyl-*α*-D-glucopyranosyl uronic acid residue attached to the C-2 position of the xylose chain, and this structural type is commonly named 4-*O*-methyl-D-glucurono-D-xylan [15]. Arabinoxylans in monocotyledonous plants and cereals are mainly substituted by *α*-L-arabinofuranosyl units at C-2 or C-3 of the xylose chain but may also be substituted by acetyl groups [5]. Arabinoglucuronoxylans are linked to 4-*O*-methyl-D-glucuronyl and *α*-L-arabinosyl groups at C-2 and C-3 positions of the xylose backbone, respectively. The compound heteroxylan has (1→4)-*β*-D-xylopyranosyl as the main chain and various monosaccharides such as uronic acid group and arabinosyl as the branched chain [15]. Xylan is less abundant in dicotyledonous plants and more abundant in monocotyledonous plants and is the main component of hemicellulose in cell walls. Mannan [29,30] is a backbone composed of mannose linked by 1,4-β glycosidic bonds. Mannan is mainly divided into galactomannan [31] and glucomannan [32]. The main chain of galactomannan is mannose linked by 1,4-*β* glycosidic bonds, and the main chain of glucomannan is composed of glucose and mannose units linked by 1,4-*β* glycosidic bonds. Mannan is the main component of hemicellulose in the secondary cell wall of gymnosperm. *β*-glucans [33] are composed of *β*-D-glucopyranosyl units linked with 30% (1→3) and 70% (1→4) glycosidic bonds. It is a hemicellulose component of grain, located in the aleurone layer and endosperm of the cell wall [34,35], and is the main molecule related to cellulose microfiber during cell growth [36]. Xyloglucan [37] is composed of D-glucopyranose linked by 1,4-β glycosidic bond to form the main chain, and the side chain is linked to O-6 of D-glucopyranose. Xyloglucans can be divided into two types according to the side chain distribution, namely, two xylopyranose units linked to two glucopyranose units (XXGG) and three xylopyranose units linked to one glucopyranose unit (XXXG). Xyloglucan is abundant in dicotyledonous plants and is the most abundant hemicellulose in their primary cell walls. 

### 2.2. Hemicellulose Film

Although hemicellulose is different in source, composition, structure and properties, hemicellulose contains a large number of active hydroxyl groups in the main chain and side chain. These active groups make hemicellulose easy to modify, making it possible to prepare hemicellulose derivatives with ideal solubility, film-forming ability and hydrophobicity [38]. Unmodified hemicellulose is difficult to prepare continuous film from due to strong intermolecular and intramolecular hydrogen bonding. Therefore, small molecular substances such as sorbitol, glycerol and xylitol are added into hemicellulose as plasticizers to prepare complete hemicellulose film with oxygen barrier property (Table 2). Hemicellulose-based films have attracted much attention due to their dense macromolecular network structure, low mobility and oxygen barrier properties [39]. For food packaging, food quality preservation and environmental protection are expected properties of bioplastics. Hemicellulose has some disadvantages as a food packaging material due to its structural properties. The low degree of polymerization and a large number of hydroxyl groups make hemicellulose films easily absorb water, and the films show poor mechanical strength and barrier performance in a humid environment, so hemicellulose needs to be modified. In the review article of Zhao and Li [40], the chemical and physical modification methods of hemicellulose had been summarized to improve the film forming property, mechanical property and hydrophobic property of hemicellulose film. Furthermore, the use of polysaccharides in the preparation of nanocomposites is common. In addition to inorganic nanomaterials, natural nanomaterials such as biodegradable nanocrystalline fibers and CNFs have also been proved to be an effective method to improve the mechanical and barrier properties of hemicellulose.

Biodegradability is an important advantage of bioplastics as food packaging materials. Biodegradability refers to the ability of a material to break down after treatment and return to nature. Unlike synthetic polymers, most biopolymers can be broken down by biological activities such as fungi and bacteria to produce natural metabolites [41]. Cellulose, polysaccharides and proteins are common examples. Hemicellulose can be degraded into monosaccharide and acetic acid by hemicellulase produced by microorganisms. Xylan is the main carbohydrate of hemicellulose, and its complete degradation requires the synergistic action of multiple enzymes. Microbial xylanase catalyzes the hydrolysis of xylan into xylose in a mild reaction [42]. Gao et al. [43] prepared hemicellulose-based composite film by blending PVA and xylan and adding butane tetracarboxylic acid as a plasticizer. The mechanical properties of the composite films increased first and then decreased with the increase in the content of butanetetracarboxylic acid. The composite film containing 10% butanetetracarboxylic acid had a biodegradation rate of almost 41% after being buried in soil for 30 days. The hemicellulose-based film can be used as a biodegradable plastic for food packaging. 

**Table 2:** Oxygen barrier properties of different hemicellulose-based composite films.

Hemicellulose	Plasticizer	Oxygen Permeability (cm^3^·µm·m^−2^·24 h^−1^·0.1 MPa^−1^)	Reference
Feruloylated arabinoxylan	-	78.6	[44]
Feruloylated arabinoxylan	30% sorbitol	1.0	[44]
Arabinoxylan	9% glycerol	3.0	[45]
Glucuronoxylan	35% sorbitol	0.21	[46]
Arabino-glucuronoxylan	25% sorbitol	0.17	[47]
*O*-acetyl-galactoglucomannan	40% sorbitol	4.0	[48]
Galactomannan	29% sorbitol	18	[49]
Glucomannan	29% sorbitol	8.1	[49]

## 3. Nanocomposites and Nanoparticles

Polymer composites typically consist of a polymer matrix as the continuous phase and a filler as the discontinuous phase. Fillers are generally inorganic or organic materials with a certain geometry (flakes, fibers, particles). Common fillers include calcium carbonate, glass fiber, mica and other materials. The mechanical, barrier properties and thermal stability of polymers are improved by adding reinforcing compounds (fillers) to the polymer matrix. The filled polymer materials are widely used in daily life. However, most reinforcing materials exhibit poor adhesion at the interface, resulting in low interaction with the polymer matrix [50]. Macroscopic reinforcing materials usually contain defects, and the smaller the particles of the reinforcing compound, the less important the defects. The use of nanofillers has attracted extensive attention of researchers in the field of food packaging because nanotechnology can not only improve the intended use performance of polymers but also nanomaterials are more efficient in manufacturing, cost and recycling than other food packaging systems such as multilayer packaging [51]. 

### 3.1. Nanocomposite

**Figure 3:**
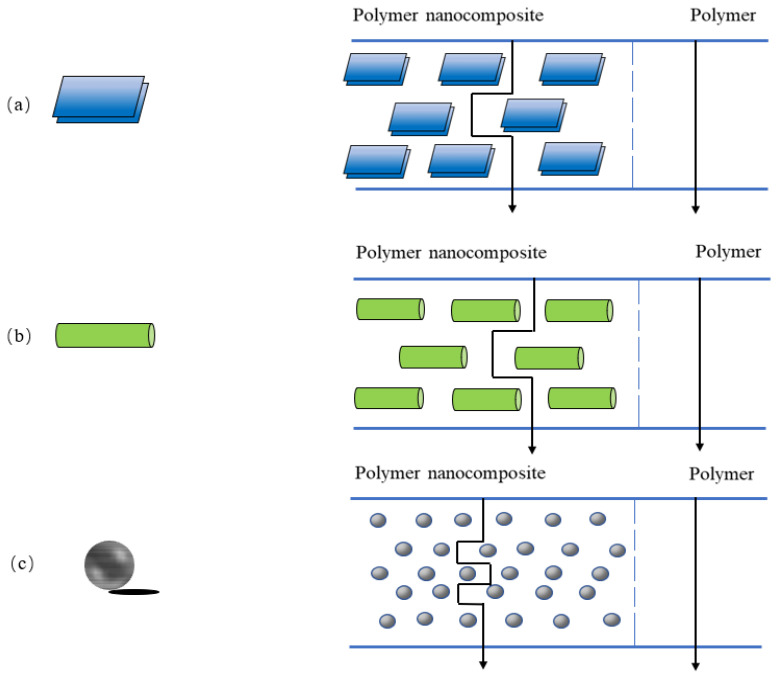
Schematic representation showing nanoparticle geometries and its corresponding tortuosity pathway in nanocomposites. (**a**) nanolayers, (**b**) nanotubes or nanofibers, (**c**) isodimensional nanoparticles.

Nanocomposite materials are composed of two or more solid phases, and at least one dimension of the dispersed particles is of nanometer scale. Nanoparticles can be classified according to their nanometer size and geometry: there are nanolayers (Figure 3a) with only one dimension on the order of nanometers, nanotubes or nanofibers (Figure 3b) with two dimensions on the order of nanometers and three-dimensional isodimensional nanoparticles [52] with three dimensions on the order of nanometers (Figure 3c). Adding nanofillers to polymers for food packaging creates a tortuous path to improve the barrier properties of packaging materials. Examples of common nanofillers used are layered silicates and cellulose nanocrystals due to their high aspect ratio and environmental friendliness [53]. The presence of nanofillers with different geometries forces vapor and gas molecules to take a more tortuous path (Figure 3), which increases the effective path length of gas molecules through the film [54], resulting in a significant lag time, thus improving the barrier properties of food packaging materials. Under the strong interaction between nanoparticles and polymer molecules, the smaller the particle size, the stronger the binding ability with the surrounding polymer molecular chain, thus reducing the chain segment mobility and osmotic diffusivity [55]. In addition, nanofillers with small particle size and large specific surface area can be used as reinforcing agents to show good adhesion with polymer at the interface and have strong interaction with polymer matrix, thus improving the mechanical properties of polymer matrix [56]. This results in a lightweight material with a stronger packaging barrier prepared by nanotechnology [57,58]. It can protect the quality of food and prolong the freshness of fruits and vegetables during transportation and storage [8]. In addition to improving the mechanical properties and barrier properties of packaging materials, some nanoparticles can also provide antibacterial activity for packaging materials. Antibacterial packaging is a packaging material that adds antibacterial agents to delay the growth of microorganisms in food. Nanoparticles with antibacterial properties include nano-ZnO, nano-Ag, etc. Antibacterial nanocomposite films are popular because of their acceptable structural integrity, the barrier properties given by the nanocomposite matrix and the antimicrobial properties contributed by antibacterial agents [59]. ZnO-based nanocomposites have shown antibacterial effects against Gram-positive (Staphylococcus aureus) and Gram-negative (Escherichia coli) [60,61]. The antibacterial mechanism of nano-ZnO is attributed to the oxidation of the cytoplasm of bacterial cells by reactive oxygen species produced by nano-ZnO, resulting in bacterial death [8]. Ag is highly toxic to a variety of microorganisms. De Oliveira Pizzoli et al. [62] studied the role of nano-Ag in poly (lactic acid)/thermoplastic starch/gelatin films. They found that the film had a strong inhibitory effect on a wide range of fungi and bacteria. The antibacterial effect of the film depended on the release behavior of nano-Ag from packaging materials in contact with food. These advantages make nanocomposites an ideal material for food packaging [63]. Currently, nanostructured materials such as layered silicates, metal oxide nanoparticles and cellulose nanocrystals are used in food packaging materials due to their mechanical strength and barrier properties. Among them, some metal nanomaterials have antioxidant activity and antimicrobial activity, which are also very important for food packaging.

### 3.2. Nanoparticles

#### 3.2.1. Nanolayers

Nanolayers are lamellar particles with a layer thickness of one to several nanometers [64]. The filler is present in the form of sheets hundreds or thousands nanometers long. Common one-dimensional nanofillers are layered silicates including smectic clays, layered double hydroxides and graphene sheets. At present, montmorillonite (MMT) is the most studied layered silicate for preparing polymer nanocomposites for food packaging applications. MMT has a 2:1 lamellar structure in which octahedral platelets of alumina are located between two tetrahedral platelets of silica and share one oxygen atom with them [52]. MMT is a typical layered nanoparticle with nanoscale layer thickness and an aspect ratio greater than 1000 [65]. The surface of MMT is negatively charged. The interlayer anion is usually balanced by the intercalated sodium ion or calcium ion, and its existence in the clay interlayer makes MMT hydrophilic, making it difficult to be uniformly dispersed in organic polymer. However, the layered silicate can be organically modified to obtain clay (organic clay) compatible with polymer [52].

#### 3.2.2. Nanofibrous Particles

Nanofibrous particles have two dimensions on the nanometer scale, typically less than 100 nm in diameter, and a larger third dimension, presenting an elongated structure [66] with an aspect ratio of at least 100. Nanotubes and nanocellulose matrices belong to the class of nanofibrous particles, which have been extensively studied as reinforcing fillers [67]. Cellulose is a natural structural material in which microfibers are linked together to form cellulose fibers. CNFs can be obtained from cellulose fibrils or microcrystalline cellulose. Currently, the two main types of nanoreinforcements obtained from cellulose are cellulose microfibers and cellulose nanocrystals (CNCs) [68]. Microfibers have size diameters 2~20 nm with a length in the micrometer range [69]. Nanocrystals have a length in the range of 500 nm to 1~2 μm and a diameter of about 2~20 nm, with a high aspect ratio [70]. Cellulose nanofiber particles have many advantages as reinforcements in food packaging materials, such as high stiffness, good barrier properties, low cost and biodegradability under certain conditions [71].

#### 3.2.3. Isodimensional Nanoparticles

Isodimensional nanoparticles are particles that structurally have a three-nanometer size (below 100 nm), such as spherical micelles, metals, metal oxides and ceramic nanoparticles. At present, the common equidimensional nanoparticles used in the field of food packaging are mainly SiO_2_ particles, ZnO and TiO_2_ and other metal oxides [52,64]. Amorphous nanoSiO_2_ is a stable and safe food additive with high specific surface area, low cost and good stability [72] and is widely used in food processing and preservation [73]. ZnO nanoparticles have high stability, nontoxicity and strong antibacterial properties [74]. TiO_2_ is widely used in the food industry as a pigment additive, which has the advantages of high stability, UV absorption, nontoxicity and low price. In addition, metal oxide nanoparticles are often used as photocatalysts to degrade organic molecules and microorganisms [8]. Owing to such advantages, metal oxide nanoparticles have received extensive attention in the field of food packaging.

## 4. Hemicellulose-Based Nanocomposite Film 

Hemicellulose-based nanocomposite film refers to the modification of hemicellulose by introducing nanoparticles into the organic matrix of hemicellulose. After the nanoparticles are uniformly dispersed in the continuous phase hemicellulose, there may be strong interfacial adhesion between the nanoparticles and the polymer matrix due to the high specific surface area to volume ratio of the nanoparticles. Although the mechanical properties and barrier properties of hemicellulose films prepared from different types of hemicellulose are different, the added nanomaterials can significantly improve these properties (Table 3). This method has the advantages of simple operation and mild conditions and is one of the main methods for physical modification of hemicellulose. Layered silicates, metal oxide nanoparticles and organic nanoparticles are the main nanomaterials used to modify hemicellulose.

### 4.1. Layered Silicate and Hemicellulose Nanocomposite Film

**Figure 4:**
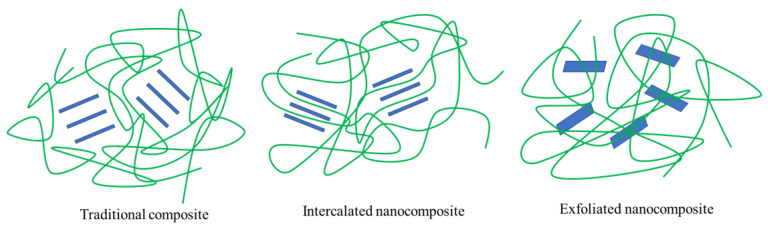
Schematic of three structures of layered silicate nanocomposites.

Three structural types of polymer-layered silicate nanocomposites can be obtained by physical mixing of polymer and silicate, depending on the nature of the polymer matrix, layered silicate, surfactant and processing conditions. One is that the lamellar structure of silicate keeps the original state, and nanoparticles are dispersed in the polymer matrix, representing the traditional composite material; the other is an intercalated structure formed by inserting a single or multiple extended polymer chains into silicate layers, forming an intercalated nanocomposite material. In a third case, well-separated clay layers are individually dispersed in a continuous polymer matrix to produce exfoliated or layered structures [96], characteristics of an exfoliated nanocomposite (Figure 4). The best performance enhancement in nanocomposites usually occurs in exfoliated structures. One of the most important improvements in polymers and biopolymer substrates used for packaging is the provision of exfoliated structures. MMT, with its small size, high aspect ratio, and large surface area, improves the zigzag path of diffused molecules, accounting for the improved barrier properties of the biopolymer (Figure 3).

**Figure 5:**
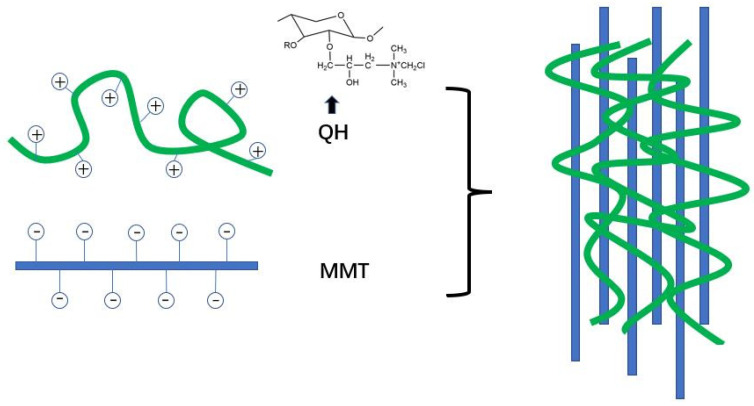
Schematic representation of organic-inorganic composite films prepared by electrostatic interaction between QH and MMT.

Guan et al. [97] prepared organic-inorganic composite film by combining positively charged quaternized hemicellulose (QH) with exfoliated anionic MMT nanosheets through electrostatic interaction and hydrogen bond in aqueous solution (Figure 5). The results showed that when the mixing ratio of QH and MMT nanosheets was 1:1, the composite film showed a smooth and uniform surface and the best thermal stability and transparency and had the potential for food packaging. In order to further improve the performance of QH/MMT composite film, Chen et al. [75] prepared hybrid film by adding PVA and chitin nanowhiskers (NCH) as reinforcing agents into QH/MMT hybrid matrix. In the process, MMT was introduced into QH solution as an inorganic phase, and PVA and NCH were used as reinforcing agents. QH and MMT were deposited in the alternating sequence of positive and negative charge layers through electrostatic interaction and hydrogen bonding to form a multilayer film with nacre structure. MMT, QH, PVA and NCH had good interfacial adhesion under strong intermolecular hydrogen bonding, which improved the strength and barrier performance of hemicellulose film.

Some researchers had prepared dense and strong nanocomposite film with nacre structure and versatility by embedding chitosan (CS) and QH into MMT [76]. The results showed that PVA and CS could improve the barrier properties of QH/MMT films (Table 3). When the mass ratio of QH: MMT: PVA was 1:1:1, the oxygen permeability of the composite film decreased from 12.26 cm^3^**·**µm·m^−2^·24 h^−1^·0.1 MPa^−1^ to 5.54 cm^3^**·**µm·m^−2^·24 h^−1^·0.1 MPa^−1^. The inorganic phase (MMT) in the composite film increased the zigzag path of diffusion molecules in hemicellulose; the PVA molecule chain increased the diffusion length along the diffusion path in the pearly layer structure and further increased the effective path length of oxygen through the film. Moreover, PVA, NCH and CS were found to improve the mechanical properties of QH/MMT composite films; a low amount of CS disturbed the arrangement of hemicellulose molecular chains between MMT nanosheets and increased hydrogen bonds, leading to a crystalline structure with less roughness than the composite films, and thus significantly improved tensile strength of QH/MMT films. Liu et al. [98] modified hemicellulose powder with organic montmorillonite (OMMT) and studied the interaction mechanism between OMMT and hemicellulose powder. The results showed that the distance between layers of OMMT was large, and the hemicellulose molecular chain was easily embedded into OMMT to form an intercalated structure. OMMT reacted with amorphous components of hemicellulose powder to form a link with carboxyl in the hemicellulose molecular chain, which improved the hydrophobicity and mechanical properties of hemicellulose better than Na-MMT. Compared with the reported unmodified hemicellulose films (Table 3), these reinforcing components with good biocompatibility and nontoxicity are beneficial to the application of hemicellulose in food packaging.

### 4.2. Inorganic Nanoparticle and Hemicellulose Nanocomposite Film

**Figure 6:**
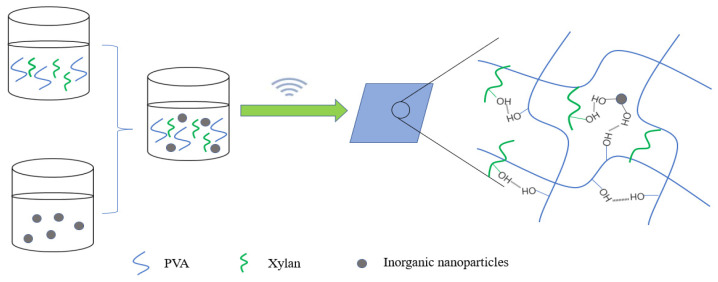
Synthesis of inorganic nanoparticles and PVA/xylan composite film.

**Figure 7:**
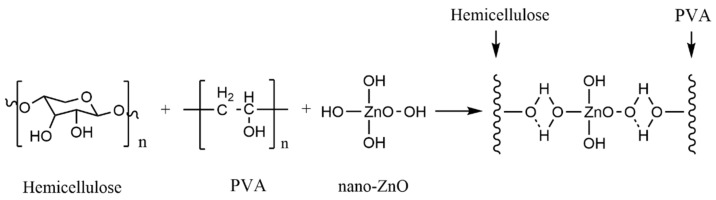
Hydrogen bond interaction between nano-ZnO particles and PVA/xylan composite film.

Commonly used inorganic nanomaterials are Ag, nano-SiO_2_, ZnO and TiO_2_. In particular, nano-SiO_2_, -ZnO and -TiO_2_ have high safety and lower cost than other nanoparticles (such as Ag nanoparticles) and have become a research hotspot of food packaging material. Liu et al. [78] prepared an organic–inorganic hybrid membrane by blending nano-ZnO and nano-SiO_2_ with PVA and xylan (Figure 6). The research proved that there was hydrogen bond interaction between the nanoparticles and PVA and xylan (Figure 7). A low dose of nanoparticles had a good dispersion effect in the polymer. Well-dispersed nanoparticles could not only promote the interfacial bonding between nanoparticles and the polymer but also reduce the free volume inside the polymer. The experimental results showed that when the content of nano-ZnO and nano-SiO_2_ was 3%, the tensile strength of the composite film increased to 20.4 MPa and 22.5 MPa, respectively, while the water vapor permeability and oxygen permeability reached the minimum value (Table 3). Nano-ZnO and nano-SiO_2_ can effectively improve the mechanical strength and barrier property of the composite film.

Organic films have low density and high flexibility. Inorganic particles mixed into organic films not only play a strengthening and barrier effect but also make the composite film have the unique properties of inorganic particles. Food packaging materials should have a shielding effect against incoming and outgoing ambient gases and ultraviolet light [99]. Some nanofillers can act as ultraviolet shields and absorb harmful radiation [100]. Nano-TiO_2_ has the advantages of high stability, nontoxicity, low cost, photocatalytic activity and UV shielding. It can be divided into rutile nano-TiO_2_ and anatase nano-TiO_2_ according to its crystal form. Rutile TiO_2_ is widely used in the fields of food packaging and wastewater treatment because of its high scattering effect and good shielding effect against ultraviolet light. Ren and colleagues [79] studied the influence of different crystal forms of TiO_2_ on the performance of PVA/xylan composite film and found that nano-TiO_2_ made the composite film have higher ultraviolet shielding performance, and the ultraviolet transmittance of rutile TiO_2_ composite film was lower than that of anatase TiO_2_, which was attributed to the high scattering effect of rutile TiO_2_. In addition, the tensile strength of the composite film with 2% rutile TiO_2_ was 30.73 MPa, which was much higher than that of pure PVA/xylan composite film and anatase TiO_2_ composite film. The results showed that intermolecular hydrogen bonds were formed between PVA/xylan and TiO_2_ at low content, but with the increase in TiO_2_ content, the nanoparticles were agglomerated in the polymer matrix and the dispersion effect was poor, which led to the poor interfacial adhesion between TiO_2_ and PVA/xylan matrix and the decline in mechanical properties of composite films. In addition, Zhang et al. [80] also studied the effect of nanoTiO_2_ on the antibacterial activity of hemicellulose/chitosan composite film. A circular film 15 mm in diameter was placed on an agar plate cultured with Escherichia coli and Staphylococcus aureus and left at 37 °C for 12 h. The results showed that with the increase in TiO_2_ content, the growth inhibition of the film on Escherichia coli and Staphylococcus aureus increased. This is attributed to the generation of reactive oxygen species by TiO_2_, which can inhibit bacterial growth by oxidizing polyunsaturated phospholipids in cell membranes [101].

Due to their high surface energy, inorganic nanoparticles have a strong aggregation tendency in aqueous solution, which will reduce the efficiency of improving the performance of polymers. Therefore, using silane coupling agents is one of the common methods to improve the dispersibility of inorganic nanoparticles in polymers. Liu et al. [81] had studied the modification of nano-TiO_2_ [102] with γ-aminopropyltriethoxysilane (KH550) so as to reduce the surface energy of nano-TiO_2_ and improve its dispersibility in polymer. TiO_2_-KH550 was added into PVA/xylan composite film, and hydrogen bonds were formed between well-dispersed TiO_2_-KH550 nanoparticles and the organic polymer, resulting in a physical cross-linking structure and strong interaction, forming dense film. At a dosage of 1.5% TiO_2_-KH550, the composite film reached the maximum tensile strength (27.3 MPa), the minimum water vapor permeability and the minimum oxygen permeability (Table 3). The tensile strength of TiO_2_-KH550 blended PVA/xylan composite film was 70% higher than that of pure PVA/xylan composite film, and the permeability of water vapor and oxygen were 31% and 41% lower than that of pure PVA/xylan composite film, respectively. The ultraviolet transmittance of the composite film at 400 nm was almost zero. The nanocomposite film could protect food from the oxidation effect of ultraviolet light. Composite films prepared by mixing inorganic nanoparticles with organic polymers have better properties than organic films [103]. The hemicellulose composite film doped with inorganic nanoparticles has certain application potential in food packaging.

### 4.3. Organic Nanomaterials and Hemicellulose Nanocomposite Film

#### 4.3.1. Nanocellulose and Hemicellulose Film

In addition to inorganic nanomaterials, organic nanomaterials have been widely used for polymer properties enhancement in the field of food packaging. Nanocellulose is a typical organic nanomaterial. Cellulose nanomaterials, as a green nanofiller, can also effectively improve the performance of polymer [104]. Such green nanomaterials include nanocellulose fibers and nanocellulose whiskers. Peng et al. [82] prepared hemicellulose-based nanocomposite films with enhanced mechanical properties by blending CNFs with xylan-rich hemicellulose (XH). The addition of 5% CNFs could obtain continuous and complete films, and CNFs significantly improved the film-forming properties of XH-based films. Due to the high aspect ratio and mechanical strength of CNFs and the strong interaction between CNFs and XH matrix, the mechanical properties of XH-based composite films were enhanced, which provided an efficient and simple method for preparing high strength hemicellulose-based films.

Saxena and colleagues [105] prepared xylan/sorbitol composite film with good strength and barrier properties by using nanocellulose whisker (NCW) as the reinforcing agent. It was found that the X-ray diffraction peaks of xylan-NCW nanocomposite films changed from weak and broad peaks to sharp peaks with the increase in NCW content. When the content of NCW was 50%, the crystallinity index of the composite film increased from 26% to 61% compared with that of the film without NCW. There was a significant interaction between the xylan film and NCW, which improved the crystallinity of the nanocomposite film matrix. With the addition of NCW, the amount of xylan adsorbed on cellulose increased, the content of crystalline substances in nanocomposites increased and the permeability of water molecules into the film decreased. The high crystallinity in the semicrystalline polymer system improved the strength and barrier properties of the xylan nanocomposite film. Pereira et al. [83] added CNCs to wheat straw hemicellulose to prepare biodegradable hemicellulose-based composite films. Hydrogen bonding interaction between CNCs and the matrix was found to enhance the mechanical strength of hemicellulose films. CNCs had the characteristics of high strength and insolubility in water and high crystallinity; this allowed water vapor to pass through the film in a curved path (Figure 3), thus improving barrier properties of the hemicellulose film to meet barrier requirements required for food packaging materials. Bao et al. [84] added NCW into chitosan/xylan matrix and successfully prepared chitosan-xylan/NCW nanocomposite film, combining the antibacterial property of chitosan, antioxidant property of xylan and good mechanical property of NCW. The results showed that the tensile strength and elongation at break of the nanocomposite films increased significantly with the addition of 12% NCW, and the swelling ratio decreased with the increase in NCW content. In addition, the nanocomposite film had an inhibitory effect on Staphylococcus aureus and Escherichia coli. Chitosan could change the permeability of cell membrane through the interaction of polycationic amino and anionic substances on the surface of bacterial cells and formed a thin film on the surface of cell membrane to prevent nutrients from entering cells [106,107]. Therefore, the antibacterial activity of the nanocomposite film mainly came from chitosan. The antioxidant activity of the film components was determined by the ABTS method [108]. The ABTS^+^ radical was generated by a mixture of ABTS (2,2′-azino-bis (3-ethylbenzothiazoline-6-sulfonic acid)) and K_2_S_8_O_2_ placed under the dark for 12 h. The ABTS^+^ scavenging activity of the xylan reached 94.1%, which indicated that the xylan had antioxidant effect. The nanocomposite film has application potential as a packaging material for fruits and vegetables.

#### 4.3.2. Other Organic Nanoparticles and Hemicellulose Blend Film

Wang et al. [85] prepared a curcumin-loaded konjac glucomannan (KGM)/zein edible nanofibers(ZNs) film using electrospinning technology. The results showed that the addition of ZNs resulted in hydrogen bonding interaction between KGM and zein, which improved the thermal and hydrophobic properties of the hemicellulose films. The water contact angle of the films increased from 7.5° to 57.5°. Curcumin, as an antioxidant and an antibacterial agent, could cause cell membrane permeation of staphylococcus aureus and Escherichia coli, so it was determined the KGM/zein/curcumin composite nanofiber films had good antibacterial effect on Escherichia coli and Staphylococcus aureus. The composite nanofiber film had antibacterial (inhibition zone of 12~20 mm) and antioxidant (scavenging activity increased by about 15%) functions, which were beneficial to its application in active food packaging materials. 

Wu et al. [86] conjugated CS/gallic acid (GA) nanoparticles (CGNPs) to a KGM matrix by solvent casting. The introduced 5~10% CGNPs were uniformly dispersed in the KGM film matrix and formed a hydrogen bond interaction with the polymer matrix so that the free volume of the composite matrix was reduced and the mechanical and barrier properties of the biological nanocomposite film were obviously improved. The KGM/CGNPs biological nanocomposite film had good antibacterial activity against food-borne pathogens such as Gram-positive Staphylococcus aureus and Gram-negative Escherichia coli. In addition to the antibacterial properties of CS, GA could also destroy the outer membrane of bacteria through metal ion chelation, resulting in bacterial death. The antibacterial activity of hemicellulose nanofilms containing CGNPs against Escherichia coli was weaker than that against Staphylococcus aureus, which might be related to the difference of microbial cell wall structure. With the addition of CGNPs, the antibacterial activity of the bio-nanocomposite film was enhanced. The film has a wide application prospect as a fruit packaging material in food packaging. 

Researchers have optimized the comprehensive performance of hemicellulose-based film by mixing hemicellulose with nanomaterials. The mechanical strength of hemicellulose composite films with inorganic nanomaterials was mostly more than 20 MPa, while the mechanical properties and water vapor barrier properties of hemicellulose composite films were improved by organic nanoparticles. The addition of nanomaterials increased the tensile strength of the hemicellulose film to 43.5 MPa and decreased the oxygen permeability to 2.24 cm^3^**·**µm·m^−2^·24 h^−1^·0.1 MPa^−1^. Compared to conventional films such as EVA, LDPE and HDPE (Table 3), the barrier property and tensile strength of the hemicellulose-based nanocomposite film have advantages but poor flexibility. In addition, some nanoparticle may provide antibacterial properties, oxygen scavenging properties, ultraviolet shielding properties, etc., to food package materials. Nanomaterials with antibacterial and antioxidant activities are beneficial to the application of hemicellulose nanocomposite films as packaging materials for fruits and vegetables. Hemicellulose-based nanocomposite films can not only protect food from environmental factors but also incorporate the characteristics of nanomaterials, so hemicellulose-based nanocomposite films have broad prospects for development in degradable food packaging. 

So far, hemicellulose-based nanocomposite film has been initially applied in the field of food packaging (Table 4). Lei et al. [109] used MMT and alkyl ketone dimer as functional aids to prepare a hemicellulose/MMT nanocomposite film for green asparagus preservation. The results showed that MMT and alkyl ketone dimer could synergistically improve the barrier and hydrophobic properties of hemicellulose films at a relative humidity of 86%. The static contact angle of the nanocomposite film increased by 131.9%, and the water vapor permeability decreased by 72.2%. In addition, alkyl ketone dimer could also improve the respiratory barrier performance of the film in a high-humidity environment. The hemicellulose-based nanocomposite film could effectively delay the loss of nutrients such as soluble protein and vitamin C in the green asparagus and prolong the shelf life of the green asparagus from 4 days to 7 days. Zhang et al. [110] studied the effect of nano-SiO_2_ on the performance of KGM/carrageenan composite film and tested the storage quality of white mushrooms packaged with this nanocomposite film. It was found that there was a strong intermolecular hydrogen bond between nanosilica and KGM/carrageenan. The film with a 0.3% nano-SiO_2_ addition had the best mechanical properties and barrier properties. The Si−O bond in the KGM/carrageenan/nano-SiO_2_ film could affect the absorption, dissolution, diffusion and release of CO_2_/O_2_. The internal atmosphere was changed by increasing CO_2_ and reducing O_2_, which reduced the respiration of white mushrooms and slowed down the metabolic activity. This film could extend the shelf life of white mushrooms from 5 days to 12 days and maintain the whiteness, appearance and hardness of mushrooms. In addition, Louis et al. [111] also prepared hemicellulose-reinforced film using nanocellulose, starch and nano-hemicellulose. The tensile strength of the film increased from 1.23MPa to 5.45MPa. At the same time, the nanocomposite film was superior to the film without nanoparticles in maintaining the pH value, color and hardness of mushrooms and could maintain the quality of mushrooms for up to 6 days.

## 5. Outlook

Development of food packaging materials with advanced functions has been receiving constant research attention. Hemicellulose-based nanocomposite films prepared by blending with nanoparticles were found to significantly improve the comprehensive properties In recent years, the research on hemicellulose nanocomposite films mainly focused on mechanical, barrier and thermal property enhancements by using nanoparticles with different geometric shapes. However, other properties including UV blocking and antibacterial effect have also emerged.

In light of nanoparticle aggregation, use of surfactants to organically modify nanomaterials for improved dispersion may be considered, and the safety of nanomaterials as food packaging additives should be taken into consideration.

Current antibacterial agents in hemicellulose films are mainly organic. Metal nanoparticles are expected to withstand more stringent processing conditions. In choosing antimicrobial agents, attention should be paid to metal/metal oxide nanoparticles on hemicellulose films. In addition, the impact of nanocomposites on human health is of primary concern since nanoparticles may be released from food packaging materials. Modification of hemicellulose with natural, edible and digestible polysaccharide organic nanoparticles could be an area of interest. In addition to the common nanocellulose, nanomaterials derived from starch or chitin are also considered valuable in the development of wholly green hemicellulose-based films for food packaging.

## Figures and Tables

**Table 1:** Source, solvent and molecular weight of representative hemicelluloses.

Hemicellulose	Source	Solvent	*M*_w_ (g/mol)	Ref.
*O*-Acetyl-4-*O*-methyl-glucurono-xylan	Hardwood	Water or alkaline solution	5000~130,000	[15,17]
*β*-glucans	Oat	Water	1,029,000~1,589,000	[18]
Arabino-4-*O*-methyl-glucurono-xylan	Softwood, grain straw	Water	30,000~370,000	[15,19]
Glucomannan	Hardwood, konjac	Alkaline solution	20,000~60,000	[20,21]
Xyloglucan	Tamarind seed	Water	1,500,000~2,000,000	[22]
Galactomannan	Delonix regia seed	Water	580,000	[23]
Arabinoxylan	Rye	Water or alkaline solution	443,000~556,000	[24]

**Table 3:** Mechanical properties and barrier properties of different films.

Films Composition	Reinforcing Agents	TS * (MPa)	EAB ** (%)	OP *** (cm^3^·µm·m^−2^·24 h^−1^·0.1 MPa^−1^)	WVP **** (10^−11^·g·s^−1^·m^−1^·Pa^−1^)	Ref.
MMT/QH	1 wt% MMT	19.8	0.5	12.26	-	[75]
NCH blended MMT/QH	1 wt% MMT	24.2	1.7	44.41	-	[75]
PVA blended MMT/QH	1 wt% MMT	31.4	1.1	5.54	-	[75]
CS blended MMT/QH	2 wt% MMT	43.5	3.2	11.16	31.9	[76]
Xylan–alginate	-	8.87	51.29	-	39.4	[77]
Nanoclays blended xylan	5 wt% bentonite	18.86	46.7	-	20.1	[77]
PVA/xylan	-	-	-	6.82	3.97	[78]
Nano-ZnO blended PVA/xylan	3 wt% nano-ZnO	20.4	-	5.28	3.14	[78]
Nano-SiO_2_ blended PVA/xylan	3 wt% nano-SiO_2_	22.5	-	5.003	3.03	[78]
Nano-ZnO blended HC/PVA	1 wt% nano-ZnO	-	87.18	2.24	38.9	[11]
PVA/xylan	-	23.54	327.23	-	4.3	[79]
Nano-TiO_2_ blended PVA/xylan	2 wt% nano-TiO_2_	30.73	192.91	-	3.46	[79]
Chitosan/hemicellulose	-	13.72	30.49	-	44.0	[80]
Nano-TiO_2/_CS/hemicellulose	20 wt% nano-TiO_2_	25.75	19.95	-	28.8	[80]
PVA/xylan	-	16.1	-	6.82	3.97	[81]
TiO_2_-KH550/PVA/xylan	1.5 wt% TiO_2_-KH550	27.3	-	4.013	2.75	[81]
CNFs-reinforced hemicellulose	15 wt% CNFs	28.9	1.8	-	-	[82]
Hemicellulose	-	8.71	3.75	-	0.12	[83]
CNCs-reinforced hemicellulose	8 wt% CNCs	14.98	2.36	-	0.071	[83]
Chitosan/xylan	-	4.9	6.47	-	-	[84]
CS-xylan/NCW	12 wt% NCW	16.04	11.49	-	-	[84]
Konjac glucomannan	-	4.25	-	-	-	[85]
Curcumin/KGM/ZNs	ZNs	7.34	-	-	-	[85]
KGM	-	30-35	42.23	-	18.22	[86]
KGM/CGNPs	10 wt% CGNPs	35~45	26.61	-	10~15	[86]
Xylan/lignin	-	1.39	56.76	-	-	[87]
Banana flours film	-	23.4	8.3	-	24.9	[88]
Quaternized hemicellulose	-	10.02	1.28	-	-	[89]
EVA	-	17.04	912	145.8	-	[90]
LDPE	-	15.18	289	78.2	-	[90]
HDPE	-	20.29	553	24	-	[90]
PET	-	45	335	58.34	-	[91]
EVOH	-	40	230	2.77	-	[92,93,94,95]

* TS: Tensile strength; ** EAB: Elongation at break; *** OP: Oxygen permeability; **** WVP: Water vapor permeability.

**Table 4:** Application of hemicellulose-based nanocomposite film in food packaging.

Film	Food	Packaging Effects	Ref.
Hemicellulose/montmorillonite	Green asparagus	Delayed the loss of soluble protein, vitamin C and other nutrients; Extended the shelf life to 7 days	[109]
KGM/carrageenan/nano-silica	White mushrooms	Improved the quality of food preservation; Extended the shelf life to 12 days	[110]
Nanocellulose/nanohemicellulose/starch	Agaricus bisporus	Retained the quality of mushrooms; Extended the shelf life to 6 days	[111]
Hemicellulose/polyethylene/nano-silver	Vegetable	Extended preservation time	[112]

## Data Availability

Not applicable.
